# Supplementary material for: Anomalous non-equilibrium response in black phosphorus to sub-gap mid-infrared excitation
Source: Nat Commun. 2022 May 13;13:2667. doi: 10.1038/s41467-022-30341-4 (PMC9106664; doi:10.1038/s41467-022-30341-4)
Supplement: Supplementary file 1 — Supplementary information [file 41467_2022_30341_MOESM1_ESM.pdf]

Anomalous non-equilibrium response in black phosphorus to sub-gap mid-infrared excitation

Angela Montanaro[1,2], Francesca Giusti[1,2], Matteo Zanfrognini[3], Paola Di Pietro[2], Filippo Glerean[1,2], Giacomo Jarc[1,2], Enrico Maria Rigoni[1,2], Shahla Y. Mathengattil[1,2], Daniele Varsano[4], Massimo Rontani[4], Andrea Perucchi[2], Elisa Molinari[3,4], and Daniele Fausti[1,2,*]

[1]*Department of Physics, Università degli Studi di Trieste, 34127 Trieste, Italy*
[2]*Elettra Sincrotrone Trieste S.C.p.A., 34127 Basovizza Trieste, Italy*
[3]*Dipartimento FIM, Università degli Studi di Modena e Reggio Emilia, 41125 Modena, Italy*
[4]*Consiglio Nazionale delle Ricerche - Istituto Nanoscienze, 41125 Modena, Italy*

[*] *Correspondence: daniele.fausti@elettra.eu*

SUPPLEMENTARY INFORMATION

Supplementary Note 1: Static characterization via temperature-dependent FTIR measurements

We characterized the sample by performing steady-state reflectivity measurements in the far- and mid-infrared spectral range at different temperatures. The measurements were performed at the SISSI infrared beamline [1] of the Elettra synchrotron in Trieste (Italy). Reflectivity data were collected using a Bruker Vertex 70v interferometer. The reflectivity is rather flat above 4000 cm^{-1} and reaches a value close to 0.3 for all the temperatures examined (Supplementary Fig. 1a). In the mid-infrared region, from 500 to 4000 cm^{-1}, we observe an increase in the reflectivity which features a mild dependence on the sample temperature. In the far-infrared range, the reflectivity is dominated by a peak localized at ~130 cm^{-1}, that we assign to the B_{1u} IR-active optical mode, in agreement with previous studies [2-5].

From the reflectivity data, we retrieved the optical conductivity for the whole set of temperatures through the Kramers-Kronig relations, as shown in Supplementary Fig. 1b. On top of the phonon mode (more prominent at low temperatures), we reveal a Drude-like contribution in the low-frequency range, due to the presence of free charge carriers. At higher frequency, a significant rise in the conductivity marks the gap energy of the sample at approximately 2250 cm^{-1} (~280 meV). The edge clearly shifts towards higher frequencies as temperature increases, confirming the anomalous temperature-dependence of the band gap that has been widely reported [6-10]. Our results are in good agreement with previous optical studies (see ref. [11] and references therein, and refs. [8,12]), although we observe a less sharp step-like edge, which is probably due to the use of unpolarized light in our measurements.

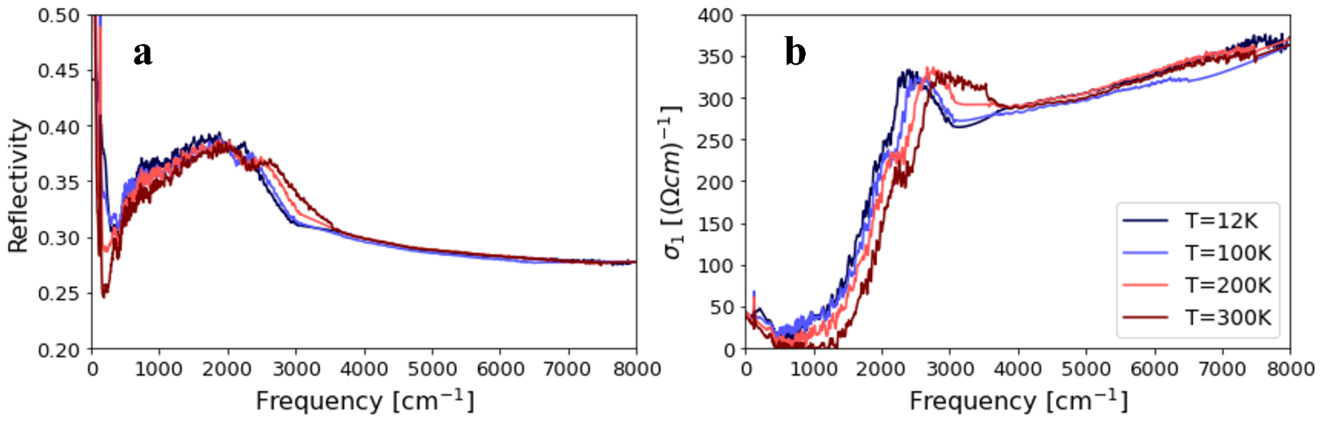

Supplementary Fig. 1: FTIR static measurements. a Steady-state reflectivity measurements for a variety of temperatures in the mid- and far-infrared spectral range. **b** Temperature-dependence of the optical conductivity extracted from panel a through the Kramers-Kronig relations.

Supplementary Note 2: Coherent longitudinal acoustic phonons generation in BP

Supplementary Fig. 2a shows the time- and spectrally-resolved relative reflectivity induced by a 3.1 eV photo-excitation for pump-probe delays up to 100 ps. After the initial photo-bleaching discussed in the main text, at longer time scales, the reflectivity is modulated by oscillations, whose frequency increases with increasing probe photon energies. This is better highlighted in Supplementary Fig. 2b where we plot pump-probe traces for selected probe photon energies averaged over 70 meV. We attribute this energy-dependent modulation of the reflectivity to the onset of Coherent Longitudinal Acoustic Phonons (CLAP) in bulk BP. CLAP detection via pump-probe spectroscopy is a largely studied phenomenon and a well-established tool to measure the longitudinal sound

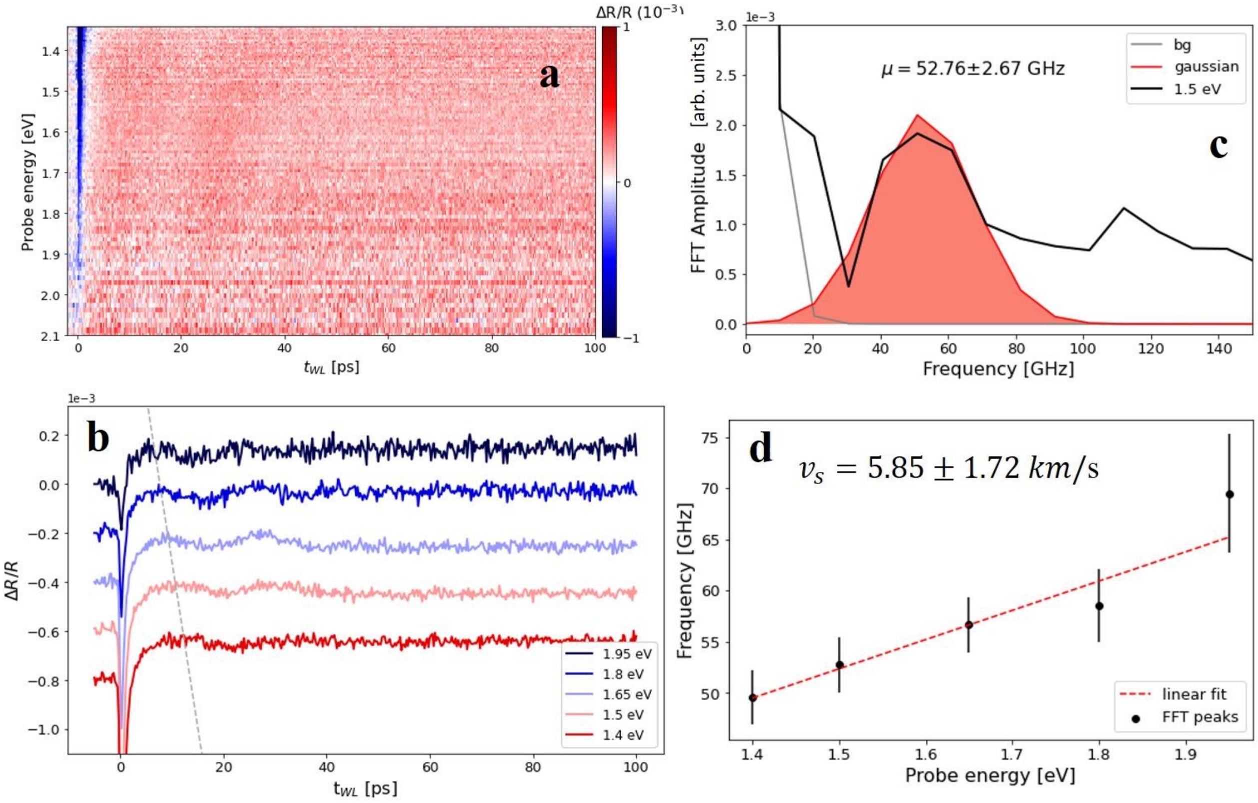

Supplementary Fig. 2: Longitudinal sound velocity revealed by Coherent Longitudinal Acoustic Phonon (CLAP) creation. **a** Transient change in reflectivity induced by visible ultrashort pulses as function of time-delay and spectral content of the white-light supercontinuum probe. **b** Horizontal cuts of the map in a for a selection of probe photon energies, each averaged over 70 meV (±35 meV with respect to the value indicated in the label). The traces are arbitrarily shifted for clarity. The dashed grey line is a guide for the eye to highlight the linear dependence of the CLAP frequency on the photon energy of the probe. **c** Fourier transform (black line) of the 1.5 eV curve in b. The Fourier transform calculation has been performed avoiding the early-time negative dynamics of the signal. The peak arising at about 50 GHz was fitted by a Gaussian-like function (red shape) to estimate its central frequency. **d** Central frequency of the Fourier-transform estimated as in c as function of the selected probe photon energies (black bullets). The dashed red line indicates a linear fit to the data, whose slope gives an estimation of the longitudinal sound velocity of black phosphorus through Supplementary Equation (1) [14]. Error bars indicate the uncertainty associated to the fit of the Gaussian-like function in c.

velocity in crystals [13-16]. In this framework, the pump pulse initiates a travelling strain wave which propagates away from the surface at the longitudinal sound velocity of the material and periodically shapes its dielectric function. When the probe impinges on the sample, reflection from both the surface and the CLAP oscillations will contribute to the measured reflectivity, resulting in interferential processes that cause the oscillatory behaviour observed in Supplementary Fig. 2b. Being the result of an interferential process, the oscillating frequency (f) depends on the wavelength (λ) of the probe pulses, according to the following relation [14]:

$$f = \frac{2nv_s}{\lambda}$$

Supplementary Equation (1)

where n is the refractive index and v_s the longitudinal velocity.

The technique has been applied recently to bulk BP to study how the in-plane anisotropy affects the CLAP generation [17]. Here, we will use the wavelength-dependent reflectivity oscillations to estimate the sound velocity and provide a characterization of the sample.

For this purpose, we estimate the oscillating frequency by Fourier-transforming the time-traces in Supplementary Fig. 2b. We discard in the calculation the initial transient response. An example of the Fourier analysis is given in

Supplementary Fig. 2c, where the black line is the Fourier-transform of the oscillating reflectivity measured at $h\nu$=1.5 eV. We fit the peak arising at about 50 GHz with the sum of a gaussian function (red shape) and an exponential decay to account for the incoherent contributions. By repeating the same procedure for all the time-traces in Supplementary Fig. 2b, we get the oscillating frequencies that we plot in Supplementary Fig. 2d as function of the probe photon energy. A linear fit to the data estimates the longitudinal sound velocity in our sample to be v_s = 5.85±1.72 km/s, in agreement with the literature [17,18]. In the calculation, we considered the refractive index calculated by ref. [19].

Supplementary Note 3: Transient optical response to sub-gap photo-excitation for long delay time

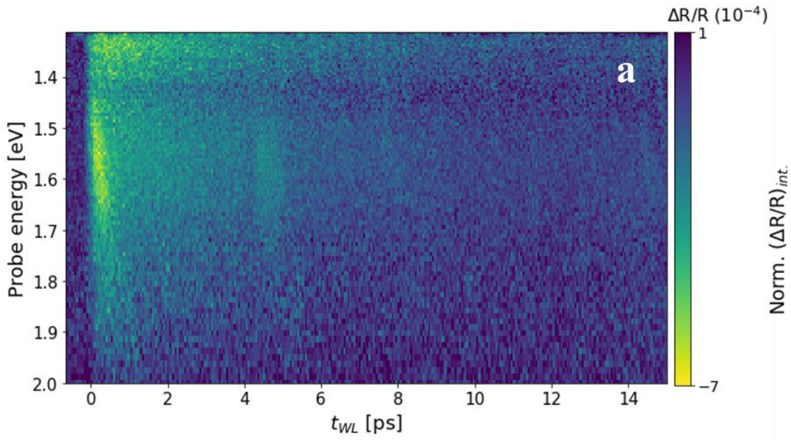

Supplementary Fig. 3: Long delay time dynamics. a Broadband transient reflectivity measured on BP at T=10 K at long delay time following a mid-infrared photo-excitation (225 meV). The pump fluence was 0.89 mJ cm^{-2}. The signal arising at $t_{WL}\simeq$ 5 ps is due to a residual reflection coming from the copper substrate and will therefore be neglected in the discussion. **b** Normalized ΔR/R spectra of the excitonic resonance averaged at different time delays, as denoted by the legend. The two traces at early times are centered at the same energy, while the trace at longer delay time is blue-shifted by ~20 meV.

We show in Supplementary Fig. 3a the time- and frequency-dependent reflectivity measured at T=10 K up to 15 ps upon photo-excitation by sub-gap mid-infrared pulses (225 meV). The fluence of the mid-infrared pump was higher than that used in Fig. 2b in the main text, and accounts for a more intense background signal on top of the excitonic resonance at ~1.6 eV. A less prominent replica of the resonance is also visible at $t_{WL}\simeq$ 5 ps and is due to a small fraction of the pump that is transmitted through the sample and reflected back by the substrate.

The temporal evolution of the optical response is the result of different many-body processes that modify both the spectral shape and the central energy of the excitonic resonance. In particular, exciton-exciton interactions and free carrier-induced band gap renormalization dominate at early times, and may result in both a transient modification in the oscillator strength and an energy shift of the exciton resonance [20]. At longer delay times (>10 ps), the interplay between the photo-excited carriers and the phonons becomes dominant. The excess energy is transferred to the lattice, which undergoes a transient temperature increase. This results in a non-equilibrium renormalization of the exciton resonance which is shifted by few meV [21].

In Supplementary Fig. 3b we show the frequency-resolved normalized spectra of the exciton at selected time delay. While the response is identical at early times (purple and green traces), the resonance is blue-shifted by ~20 meV at t_{WL}> 15 ps (yellow trace). This is consistent with the long-time dynamics of the excitonic resonances measured on other layered semiconductors, such as WSe_2 [20] and MoS_2 [22], and reinforces our assignment of the sub-gap pump-induced excitonic resonance.

Supplementary Note 4: Above-gap photo-excitation by near-infrared pulses

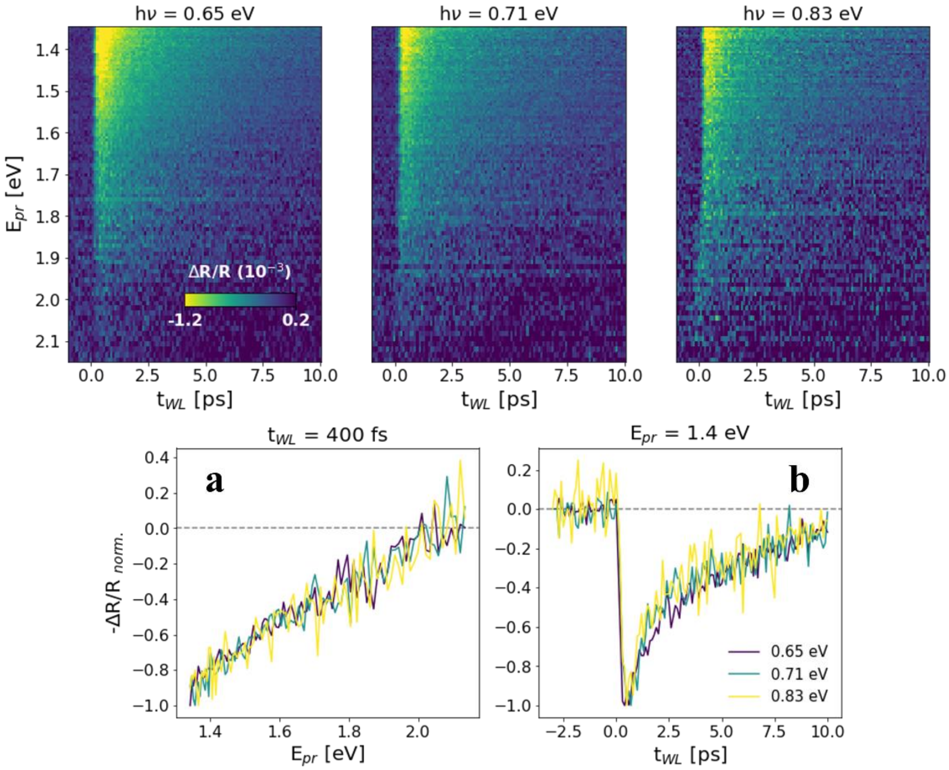

Supplementary Fig. 4: Photo-excitation by near-infrared ultrashort pulses. Transient reflectivity color-coded maps upon photo-excitation by near-infrared pulses with tunable photon energy. **a** Normalized probe energy-dependent spectra at fixed time delay (t_{WL}=400 fs) for different pump photon energy. **b** Normalized pump-probe traces at fixed probe energy (E_{pr}=1.4 eV).

In order to explore the non-equilibrium optical response upon injection of different amounts of excess energy, we photo-excited the sample with near-infrared ultrashort pulses. Considering the characteristic mid-infrared band gap of bulk BP, these photon energies are large enough to excite a photo-carrier population. At the same time, they are much lower than the visible photo-excitation discussed in the main text that may eventually initiate higher-order electronic transitions.

We used one of the two near-infrared outputs of the Twin Optical Parametric Amplifier as pump and tuned it in the range 0.65-0.83 eV. We show in Supplementary Fig. 4 the results of the experiment. There is no appreciable difference neither in the spectral shape nor in the dynamics of the signal upon photo-excitation by different photon energies (Supplementary Fig. 4a,b). The spectral dependence of the transient reflectivity is very similar to the one photo-excited by visible pulses: at early times, there is a broadband photo-bleaching (negative differential reflectivity). Similar to the optical response to 3.1 eV photo-excitation discussed in the main text, the photo-bleaching is confined below ~2 eV. Above this threshold, the optical absorption is dominated by higher-order transitions, which are not affected by Pauli blocking. However, when compared to Fig. 2a, the dynamics of the early-time photo-induced transparency due to phase space filling is slower at smaller pump photon energy. This difference could be explained as follows. The photo-bleaching by Pauli blocking is ultimately due to the fact that the probing energy levels are already occupied by the pump-excited carriers. On a picosecond time scale, intra-band scattering with phonons leads to a relaxation of the free photo-carriers that results in a reduction of the Pauli blocking contribution to the optical signal. As the pump photon energy is decreased, also the number of de-excitation channels available for the free carrier population is reduced. This could result in a slower electron-

phonon scattering dynamics and, in turn, in the observed slower dynamics of the bleach signal (Supplementary Fig. 4b).

The broadband near-infrared pump-probe measurements are a clear indication that the overall optical response of the sample to above-gap photo-excitations is similar, no matter how large the photon energy is. Only sub-gap photo-excitations trigger a suppression of the screening and unveil the exciton resonance in the monolayer.

Supplementary Note 5: Temperature-dependence of the visible and near-infrared pump-probe signal

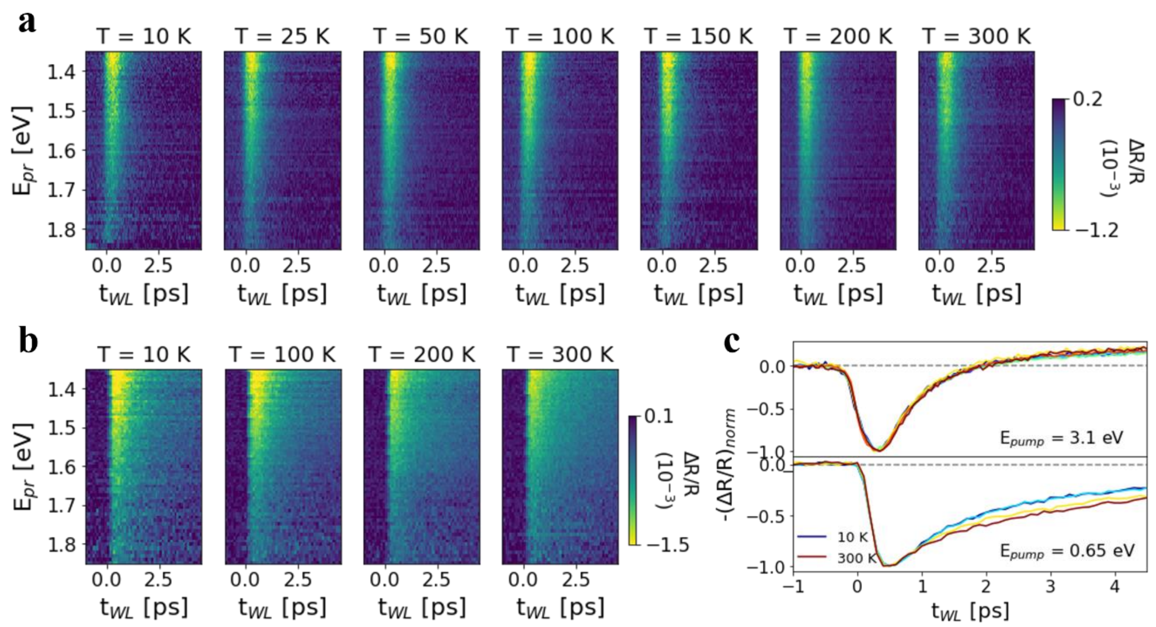

Supplementary Fig. 5: Transient reflectivity maps at different temperatures. a,b Transient change in reflectivity at different temperatures upon photo-excitation by visible (E_{pump} = 3.1 eV) and near-infrared (E_{pump} = 0.65 eV) pulses, respectively. **c** Normalized pump-probe traces at different temperatures integrated over the range 1.35-1.85 eV following a visible (top) and near-infrared (bottom) photo-excitation.

As highlighted in Supplementary Note 1, BP features anomalous thermoelectric properties. In contrast to the vast majority of semiconductors, the gap energy in bulk BP monotonically increases with increasing temperature, as also observed in our sample through FTIR measurements (Supplementary Fig. 1b). While different explanations have been proposed to describe such singular behaviour (i.e., gap-renormalization by strong electron-phonon coupling, thermal expansion [7], temperature-tunable interlayer coupling [10]), no unanimous consensus has been reached so far.

In the main text, we showed that the temperature-dependence of the electronic structure significantly affects the optical response to mid-infrared photo-excitation (Fig. 3). At fixed photon energy of the mid-infrared pulse, the photo-induced undressing of the excitonic resonance is hindered at higher temperature, where the gap energy becomes much larger than the photo-injected excess energy.

On the contrary, the optical response to visible and near-infrared pulses does not have such strong dependence on the sample temperature. We summarize in Supplementary Fig. 5 the pump-probe measurements performed on bulk BP at different temperatures upon visible (Supplementary Fig. 5a) and near-infrared (Supplementary Fig. 5b) photo-excitation. The normalized visible pump-probe traces integrated over a broad energy range almost overlap at different temperatures (Supplementary Fig. 5c, top panel). The same analysis on the reflectivity maps following a near-infrared photo-excitation (Supplementary Fig. 5c, bottom panel) shows that also in this case the overall signal is similar at different temperatures. We observe a slower decay time of the photo-bleaching at higher temperatures, possibly due to a modified electron-phonon scattering rate.

Supplementary Note 6: Fluence-dependence of the visible pump-probe signal

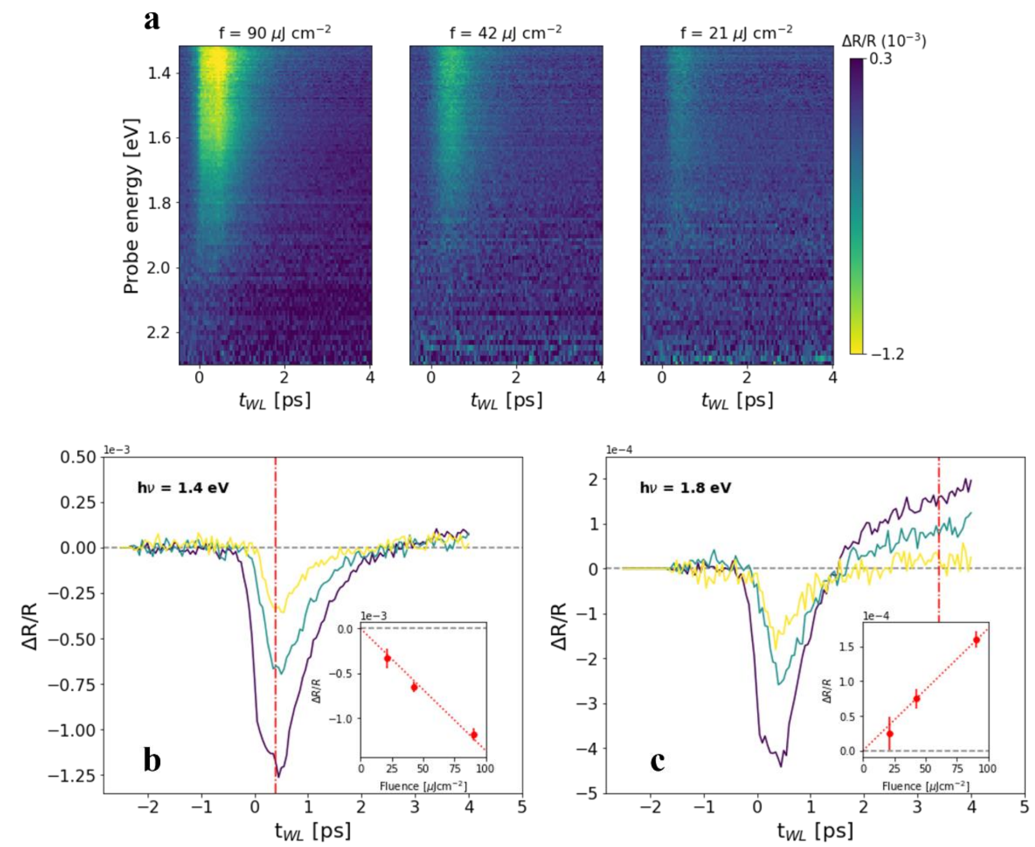

Supplementary Fig. 6: Fluence-dependent visible pump-probe measurements. a Color-coded maps of transient change in broadband reflectivity after photo-excitation by visible ultrashort pulses with variable fluence at 10 K. **b,c** Time-traces at selected probe photon energies ($h\nu$=1.4 eV and $h\nu$=1.8 eV, respectively) for all the three fluences under study. The insets show the fitted linear dependence of the transiently induced transparency at t_{WL}= 400 fs and t_{WL}= 3.5 ps. Error bars indicate the fit uncertainty.

We show in Supplementary Fig. 6 the transient reflectivity upon photo-excitation by 3.1 eV pulses with different fluence. Both the photo-induced transparency by photo-bleaching (Supplementary Fig. 6b) and the photo-induced absorption (Supplementary Fig. 6c) scale linearly in pump fluence, as shown in the insets. This is an indication that we work in a regime in which the photo-excited free carrier population is proportional to the absorbed power.

Supplementary Note 7: Fluence-dependence of the mid-infrared pump-probe signal

We analyse in Supplementary Fig. 7 the differential reflectivity following photo-excitation by MIR pulses with tunable fluence. The optical response at the lowest fluence under exam is the one discussed in the main text, where the only contribution to the signal is well localized in frequency and corresponds to the exciton resonance. When the MIR pump fluence is increased (up to almost an order of magnitude), a background signal arises on top of the exciton, whose spectral dependence (Supplementary Fig. 7b) is similar to the one measured upon above-gap photo-excitation (Fig. 2b, yellow trace). We ascribe this frequency-broad background to non-linear two-photon absorption (TPA). In order to isolate the two contributions, we integrated the differential maps in Supplementary Fig. 7a over two different energy regions: the exciton resonance lies in the range 1.45-1.9 eV, while the TPA signal dominates the low-energy side of the spectrum (1.3-1.45 eV). We plot in Supplementary Fig. 7d,e the pump-probe traces integrated over these two spectral regions and normalized over the corresponding fluences. While the exciton resonance is characterized by a fast decay of approximately 1 ps, the TPA dynamics at high fluences features a slower decay time, which is very similar to the one measured upon near-infrared (0.65 eV) photo-

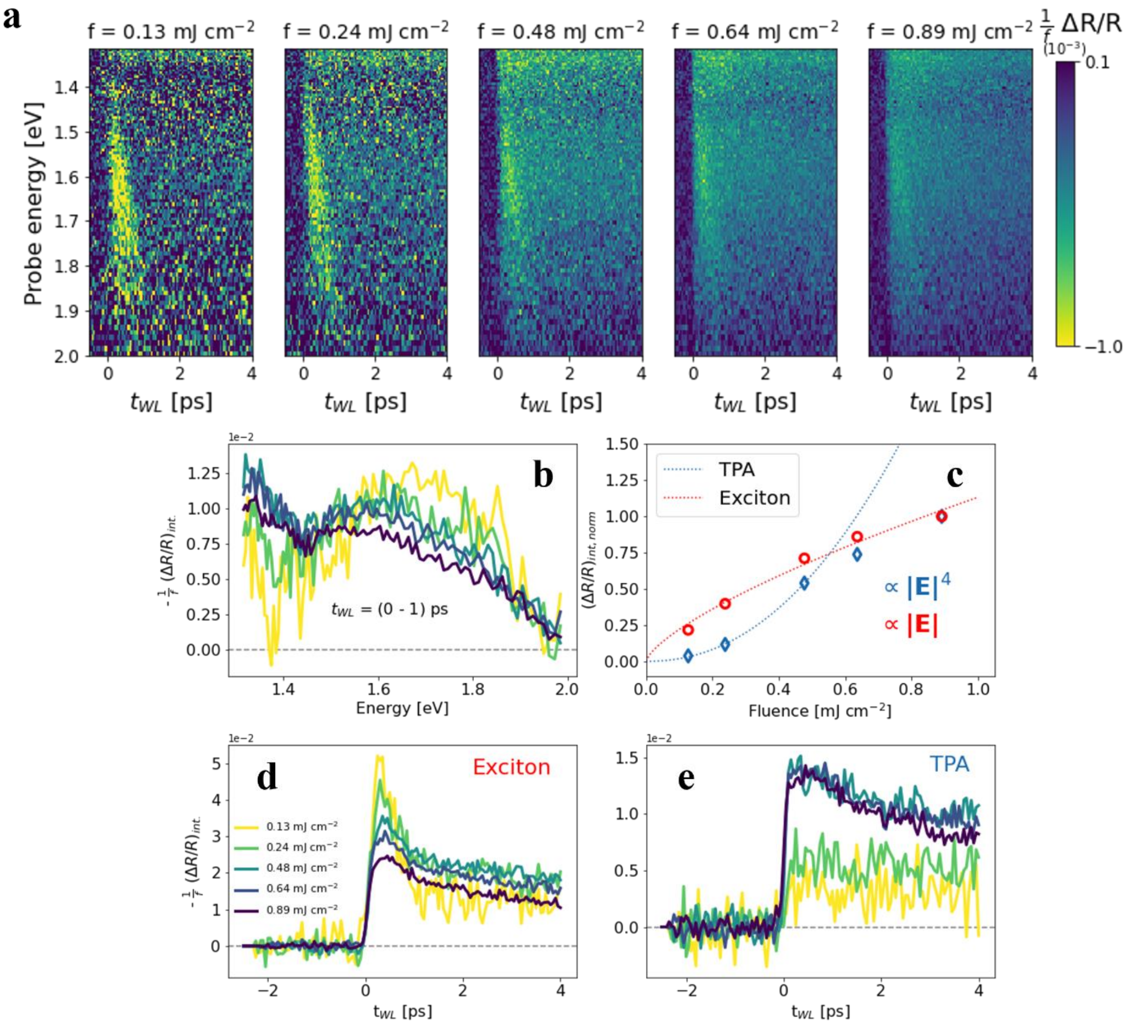

Supplementary Fig. 7: Fluence-dependent MIR pump-probe measurements. a Transient reflectivity maps at 10 K upon photo-excitation by mid-infrared (275 meV) with different fluences (f). To highlight the exciton resonance at low fluences, all maps have been rescaled by the corresponding pump intensity ($\frac{1}{f}(\Delta R/R)$). **b** Energy spectra of the maps in panel a integrated over 1 ps after the photo-excitation and normalized over the pump fluence. **c** Normalized fluence dependence of the exciton and TPA contribution. Each point in the graph is obtained by integrating the spectra in b over the region 1.45-1.9 eV for the exciton, and 1.3-1.45 eV for the TPA signal. The dotted lines are power law fits to the data. We included in the TPA fit only the three measurements at lowest fluences. **d** Time-traces integrated over the exciton resonance (1.45-1.9 eV) normalized over the corresponding pump fluence. **e** Time-traces integrated over the energy region 1.3-1.45 eV to highlight the two-photon absorption (TPA) contribution.

excitation (Supplementary Fig. 5c bottom panel). This reinforces our assignment of the TPA signal. A further confirmation comes from the fluence dependence of the exciton and TPA contributions (Supplementary Fig. 7c). We fitted the points in the plot with a power law function (βx^{α}) to extract the fluence dependence. While the TPA signal scales as the square of the fluence ($\alpha_{TPA} = 2.17 \pm 0.09$), in agreement with a two-photon process, the exciton resonance is consistent with a square root-like dependence ($\alpha_{exc} = 0.71 \pm 0.07$). This is an indication that the transient exciton absorption is the result of a coherent effect that scales with the amplitude of the electric field ($\sqrt{f} \sim |E|$). It should be noted that the two points at highest fluences of the TPA signal deviate from the expected quadratic power law. This behaviour can be explained by the saturation of optical absorption that has been observed in BP under strong illumination. Due to the Pauli blockade effect, interband transitions become forbidden and this results in a non-linear enhancement of transmittance in both few-layer and bulk BP [23].

Supplementary Note 8: Estimation of penetration depths, c-axis thermal expansion and local heating
We discuss in this section the calculation of some relevant optical quantities.

The penetration depth of the beams at a specific pump photon energy (δ_{PE}) was calculated as:

$$\delta_{PE} = \frac{1}{\alpha} = -\frac{d}{\ln(T_{PE})}$$

Supplementary Equation (2)

Where α is the absorption coefficient, T_{PE} is the measured transmissivity at the photon energy considered, and d is the thickness of the sample. Based on the measurements reported in ref. [24] and [25], we estimate the following penetration depths: δ_{3eV} =76 nm, $\delta_{0.65eV}$ =84 nm and $\delta_{275meV} = 1.5$ µm.

The interlayer spacing in bulk BP is 1.0473 nm, as measured through neutron diffraction [26]. We estimate the temperature jump required to increase the interlayer distance by 1% as:

$$\Delta T = \frac{\Delta L}{L_0}\frac{1}{\alpha_c} \simeq 850\ K$$

Supplementary Equation (3)

Where $\frac{\Delta L}{L_0} = 0.01$ and α_c is the linear thermal expansion coefficient along the stacking direction measured in ref. [27].

Finally, we estimate the local heating induced by the mid-infrared pulse (ΔT_{MIR}) as follows:

$$\Delta T_{MIR} = \frac{f(1-R)}{\delta_{275meV}\ C_{mol}}$$

Supplementary Equation (4)

Where f is the pump fluence, R is the reflectivity at 275 meV (measured in ref. [28]), and C_{mol} is the molar heat capacity (measured in ref. [29]). At the lowest mid-infrared pump fluence discussed in the main text ($f = 0.13\ mJ\ cm^{-2}$), we estimate a local temperature increase equal to about 7 K. We used in the calculation the BP density (ρ_{BP} = 2.34 g/cm^3) and the BP exact molar mass (30.97 g/mol).

Supplementary Note 9: Reflectivity maps as function of MIR pump photon energy

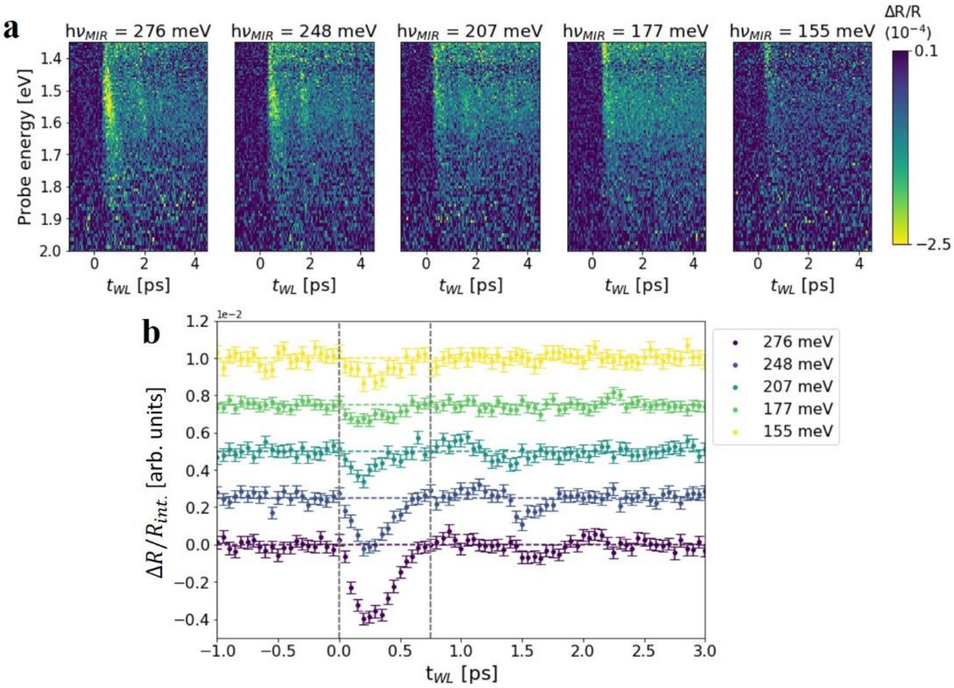

Supplementary Fig. 8: Photon energy-dependent MIR pump-probe measurements. a Transient reflectivity maps upon photo-excitation by MIR pulses with tunable photon energy at constant fluence (160 μJ cm^{-2}). **b** Pump-probe traces integrated over the spectral region (1.45-1.65) eV after the subtraction of a slow-decay component through bi-exponential fitting of the data in panel a. Error bars indicate the standard deviation associated to the energy integration.

We show in Supplementary Fig. 8a the time- and energy-resolved transient reflectivity upon photo-excitation by MIR pulses with photon energy tunable across the bulk BP band gap. The measurement at the highest MIR photon energy displays a prominent contribution of the spectral feature peaked at 1.7 eV and assigned to the lowest-energy exciton resonance in the monolayer phosphorene. A spectrally-flat background is present, along with a replica of the signal at t_{WL}~2 ps that arises from a partial reflection of the copper substrate. As the MIR photon energy is decreased, the contribution of the exciton is reduced in intensity, but the flat background is not suppressed. In order to isolate the exciton contribution and quantify it as function of the MIR photon energy (Fig. 3a in the main text), we performed a bi-exponential fit of the pump-probe traces at each probe energy. We identified a fast-decaying component associated to the exciton resonance, and a slow-decaying component that is associated to the background and that has been subtracted. We show in Supplementary Fig. 8b the energy-integrated pump-probe traces after the subtraction of the background. Fig. 3a is the result of the integration of these curves in the temporal window t_{WL}=(0-750) fs, as indicated in Supplementary Fig. 8b by the dashed grey lines.

Supplementary Note 10: Mid-infrared pump-probe signal as function of the sample temperature

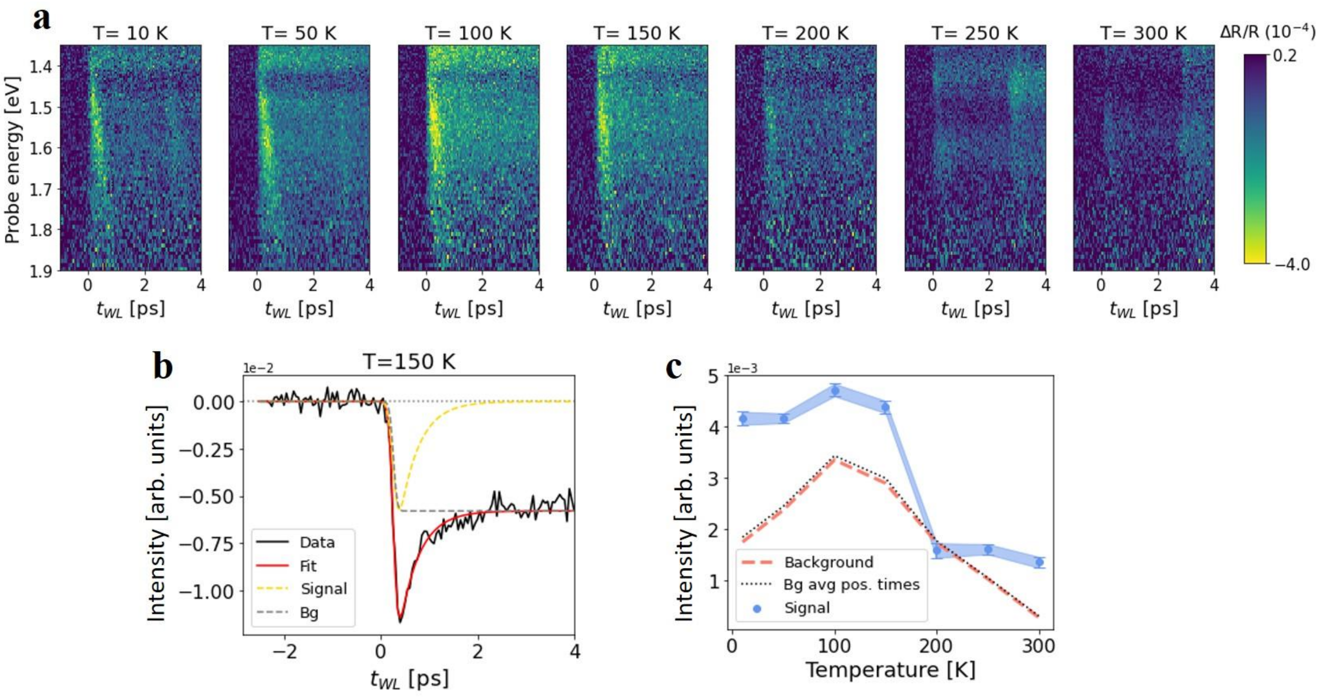

Supplementary Fig. 9: Temperature-dependent MIR pump-probe measurements. a Transient reflectivity maps upon MIR photo-excitation (hν=275 meV) at different temperatures. The fluence has been kept constant and equal to 160 μJ cm^{-2}. **b** Example at T=150 K of the analysis performed. The black curve is the pump-probe traces integrated over the spectral region 1.45-1.8 eV. The red curve is a fit to data that is the sum of a fast exponential decay (yellow curve) and a slow-decaying background component (error function, grey curve). **c** Fitted amplitude of the signal (light blue) and of the background (pink) as function of sample temperature. The dashed black curve indicates the fit-independent background obtained by integration of the pump-probe traces at positive times (3-4 ps). Error bars indicate the fit uncertainty.

We plot in Supplementary Fig. 9a the time- and energy-resolved transient reflectivity maps upon photo-excitation by MIR pulses ($h\nu$=275 meV) at different sample temperatures. The $\Delta R/R$ map at low temperature consists of the frequency-localized signal associated to the phosphorene E_{11} exciton resonance and a spectrally-flat background. Similar to the MIR photon-energy dependent measurements (Supplementary Fig. 8a), the excitonic resonance disappears at higher temperatures, while the background persists, even if reduced in intensity. In order to study the temperature dependence of the two contributions independently, we analyzed the data as follows. Firstly, we performed an integration of the pump-probe traces over the spectral range of the exciton resonance (1.45-1.8 eV) and subtracted the spurious replica of the signal at $t_{WL} \simeq$ 3 ps coming from the back-reflection of the copper substrate. The result is the black curve plotted in Supplementary Fig. 9b for a representative map at T=150 K. We fitted this curve with the sum of a fast-decaying exponential (yellow curve), which reproduces well the dynamics of the exciton, and an error function (grey curve) to fit the background. By performing the same analysis on all the maps in Supplementary Fig. 9a, we plotted in Supplementary Fig. 9c the temperature-dependent amplitude of the excitonic signal (light blue curve) and of the background (pink curve). The latter is consistent with a fit-independent integration of the pump-probe traces at positive time delays (3-4 ps, dashed black curve). The fitted amplitude of the signal (light blue curve) is the temperature-dependence at $h\nu$=275 meV discussed in the main text (Fig. 3b).

Supplementary Note 11: Contributions to calculated optical absorption

In this section, we identify the electronic transitions responsible for the absorption spectrum (computed at the independent particle level) in the range [0-3.0] eV for bulk BP. Specifically, we consider various sub-regions of the absorption spectrum and determine the pairs (*ck,vk*) of a conduction and a valence state at a given *k*-point in the Brillouin zone, for which:

- The energy difference $E_c(k) - E_v(k)$ (transition energy) is within the energy range of the considered sub-interval of the absorption spectrum;
- The dipole matrix elements $|r_{vc}^{x}|$ (computed along the *x*-direction) are the most intense.

In this way, it is possible to identify which regions of the Brillouin zone contribute to a given portion of the absorption spectrum, selecting transitions characterized by a high dipole strength.

The left panels in Supplementary Fig. 10 show the absorption spectrum computed for light polarized along the *x* direction: in each subfigure we highlight in red the portion of spectrum mainly determined by the transitions between valence and conduction states denoted by the arrows in the band structures shown in the corresponding panels on the right; the width of the arrows (denoting different transitions) is proportional to $|r_{vc}^{x}|$, normalized to the largest dipole matrix element in a given region. Further, the *k*-points, along which the band dispersions are shown, are expressed in reciprocal lattice units (rlu).

Supplementary Fig. 10a,b show that the main contribution to absorption in the range [0.3,1.0] eV comes from transitions between the last occupied valence and the first unoccupied conduction bands, with both *k* parallel to the ΓY direction in the $k_z = 0.0$ plane and with *k* parallel to the ΓZ direction.

At higher photon energies (Supplementary Fig. 10c,d, Supplementary Fig. 10e,f and Supplementary Fig. 10g,h), the absorption is explained in terms of transitions between the same valence-conduction band pairs considered before but characterized by wave vectors with progressively increasing k_z (the in-plane component remains close to Γ and mostly parallel to ΓY direction).

Looking at Supplementary Fig. 10i,l,, we can rationalize the absorption structure at about 2.2 eV as a transition between the penultimate occupied valence and the second unoccupied conduction bands, in the $k_z = 0.5$ rlu plane of the Brillouin zone, where the last two valence bands (along with the first two conduction bands) are degenerate in energy.

Finally, Supplementary Fig. 10m,n demonstrate that the main contributions to the absorption peak between 2.7 and 3.0 eV come from transitions between the penultimate valence and the second unoccupied conduction band, at the wave vectors at which the transitions responsible for the absorption in the [0.3,1.0] range occur.

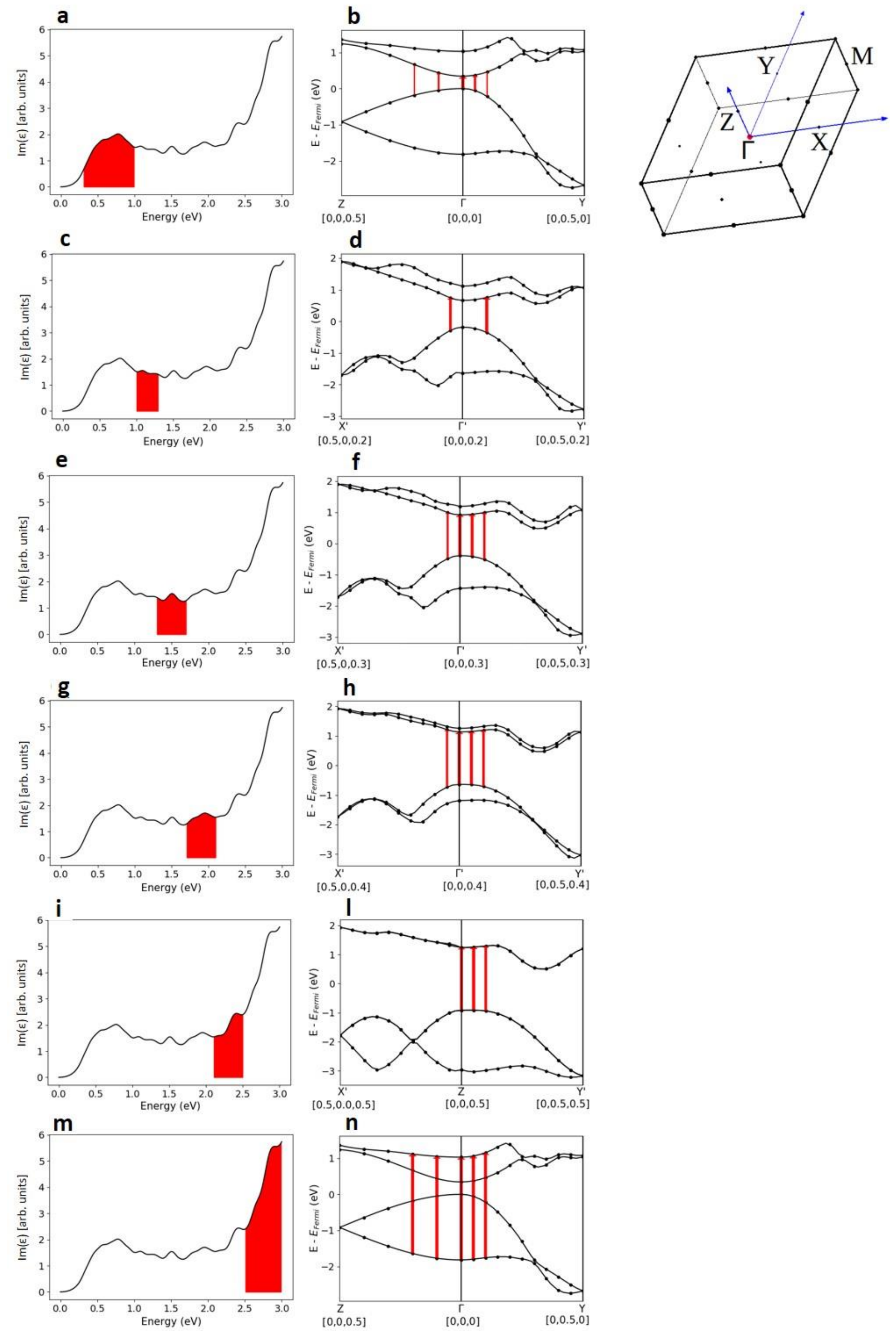

Supplementary Fig. 10: Left panels show the absorption spectrum (i.e. the imaginary part of the dielectric function) for light polarized along the *x*-axis. In the right panels, we highlight the transitions along the high symmetry directions in the Brillouin zone that are responsible for the absorption in the red-shaded area of the spectrum in the corresponding left panels. See text for details. The high symmetry points are expressed in reciprocal lattice units.

Supplementary Note 12: Hydrodynamic pressure-dependent DFT calculations

We present in this section the effect of external hydrodynamic compression on the electronic properties of BP. More precisely, we focus on applied pressure values in the range between 0.2 GPa up to 1.0 Gpa. The lattice parameters in presence of an external hydrodynamic pressure have been determined by minimizing the Enthalpy of the structure. This has been done at the DFT level using PBE exchange correlation functional. The atomic positions within the unit cell have been relaxed following the procedure outlined in 'Methods' section. Finally, the electronic properties as a function of the external pressure have been computed with Hybrid DFT, using GAU-PBE hybrid functional.

In Supplementary Fig. 11a we summarize the effect of the application of an external pressure on the lattice parameters. The resulting in-plane contraction is anisotropic: it is more prominent along the armchair direction (a), while less important along the zigzag direction (b). As discussed in the main text, the electronic band gap is significantly reduced upon the hydrodynamic compression (Supplementary Fig. 11b).

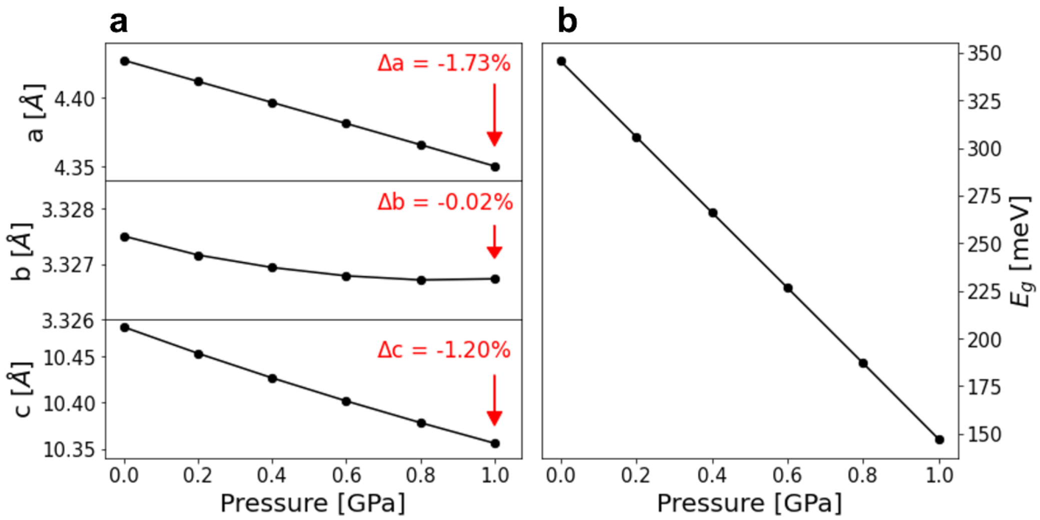

Supplementary Fig. 11: a Compression of the lattice parameters as function of the applied external pressure. We highlighted the relative changes in the lattice parameters corresponding to the highest applied pressure, as denoted by the red arrows. **b** Corresponding band gap energy plotted as function of the pressure.

Supplementary Note 13: Double-pumped pump-probe experiment

We present in this section the main results of the double-pumped time-resolved experiments that we carried out on bulk BP. In these experiments, a three-pulse scheme is adopted to study the broadband transient reflectivity of the sample in its excited state. The sample is simultaneously photo-excited by both the visible (3.1 eV) and MIR (275 meV) pumps (whose time delay τ can be arbitrarily set), and probed by the white-light supercontinuum, as depicted in Supplementary Fig. 12a. Two optical choppers placed along the optical path of the pumps and synchronized to run one at double the frequency of the other, repeatedly block the pumps, as sketched in the top of Supplementary Fig. 12a. The probe pulses (repetition rate = 5 kHz) are sorted according to the chopping scheme, so that they fall into one of the four dashed boxes in Supplementary Fig. 12a ("U" = unpumped spectra, "DP" = double-pumped spectra, "MIR" = MIR pump-excited spectra, "VIS" = visible-pump excited spectra). This differential acquisition allows to isolate, within the very same measurement, the dynamical response of the sample to just the visible pump (R_{VIS} = *(VIS-U)/U*), just the MIR pump (R_{MIR} = *(MIR-U)/U*) and the joint response to the

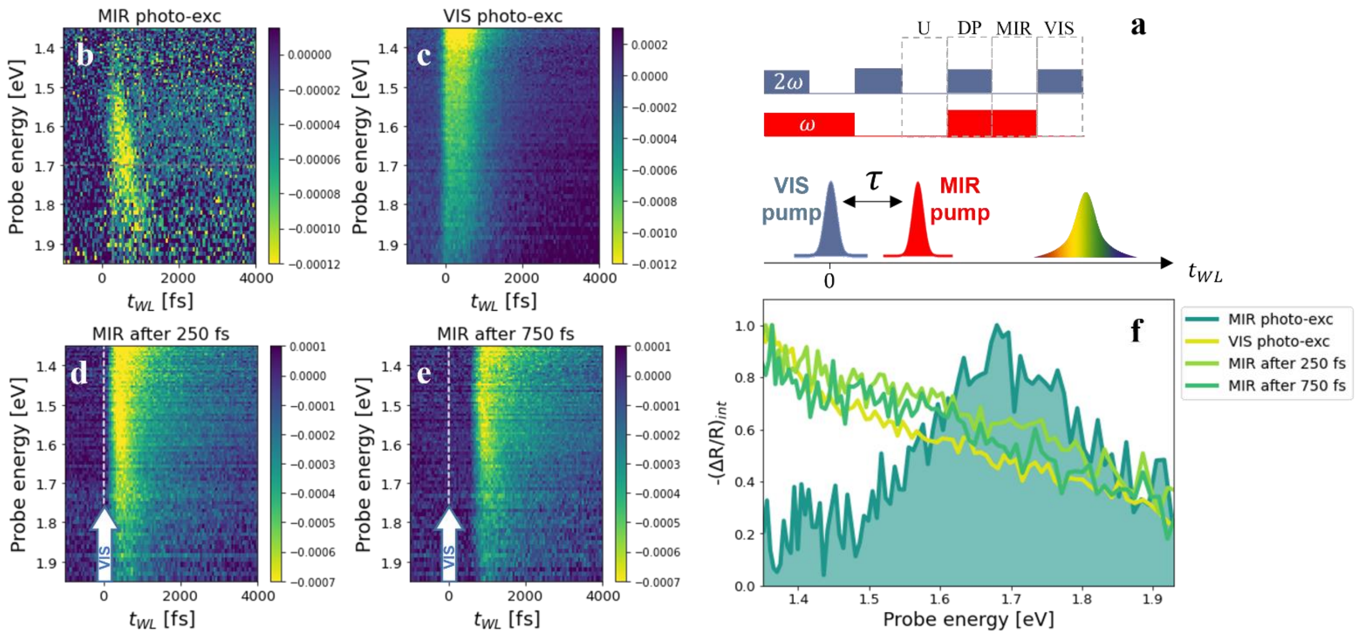

Supplementary Fig. 12: Double-pumped pump-probe measurements. a Conceptual scheme of the three-pulse experiment. The blue and red squares on the top indicate the status (ON/OFF) of the optical choppers that cut the visible and MIR pumps, respectively. The blades run synchronously (ω=45 Hz), so that the response to both the pumps (DP), to only the mid-infrared one (MIR) and to only the visible one (VIS) can be isolated. The unpumped spectra (U) are also acquired as reference, when both the choppers block the pump beams. **b,c** Transient reflectivity maps upon MIR (hν=275 meV) and visible (hν=3.1 eV) photo-excitation, respectively. **d,e** Transient reflectivity maps upon MIR photo-excitation when the sample was previously photo-excited by visible pulses. The delay between the arrival of the two pumps is 250 and 750 fs, respectively. The white arrows indicate when the above-gap photo-excitation occurs. **f** Normalized energy cuts of the maps in b-e. The spectra are averaged over 400 fs.

double photo-excitation (R_{DP} = *(DP-U)/U*). More details on the three-pulse experimental setup and the data analysis are given in ref. [30].

In Supplementary Fig. 12b,c we plot as a reference the transient reflectivity maps upon photo-excitation by MIR (R_{MIR}) and VIS (R_{VIS}) pulses, which show, respectively, the MIR-driven exciton resonance at 1.7 eV and the broadband photo-bleaching by Pauli blocking (same data discussed in Fig. 2 of the main manuscript). The strength of our three-pulse approach is the possibility to measure how the sample responds to MIR fields after a previous photo-excitation by the visible pump. The panels d,e in Supplementary Fig. 12 ($R_{DP} – R_{VIS}$) show that, if BP is previously excited by the above-gap pump at $t_{WL} = 0$ (white arrows), the MIR-driven exciton resonance is suppressed and a broadband photo-induced transparency, similar to that in Supplementary Fig. 12e, is observed. We compare in Supplementary Fig. 12f the transient spectra in the four configurations considered (maps in b-e). The suppression of the excitonic resonance in the double-pumped response reveals that the MIR-driven screening reduction is a coherent effect which takes place only in the pristine sample, where there are no free carriers. Whenever the sample is photo-excited by above-gap pulses, the transient response to MIR fields is dominated by the photo-injected carriers and the broadband Pauli blockade effect prevails. This is a further confirmation that the observed emergence of the exciton resonance is related to a non-adiabatic drive, and not to a population or excited state absorption effect.

Supplementary References

1. Lupi, Stefano, et al. Performance of SISSI, the infrared beamline of the ELETTRA storage ring. *JOSA B* **24**, 959-964 (2007).
2. Terada, Shinichi, et al. Infrared investigation of lattice vibration in black phosphorus. *Journal of the Physical Society of Japan* **52**, 2630-2633 (1983).
3. Nagahama, Toshiya, et al. Optical determination of dielectric constant in black phosphorus. *Journal of the Physical Society of Japan* **54**, 2096-2099 (1985).
4. Sugai, S., and I. Shirotani. Raman and infrared reflection spectroscopy in black phosphorus. *Solid state communications* **53**, 753-755 (1985).
5. Di Pietro, P., et al. Emergent Dirac carriers across a pressure-induced Lifshitz transition in black phosphorus. *Physical review B* **98**, 165111 (2018).
6. Baba, Mamoru, et al. Photoconduction of black phosphorus in the infrared region. *Japanese journal of applied physics* **30**, L1178 (1991).
7. Villegas, Cesar EP, A. R. Rocha, and Andrea Marini. Anomalous temperature dependence of the band gap in black phosphorus. *Nano letters* **16**, 5095-5101 (2016).
8. Ehlen, N., et al. Evolution of electronic structure of few-layer phosphorene from angle-resolved photoemission spectroscopy of black phosphorous. *Physical Review B* **94**, 245410 (2016).
9. Chen, Chen, et al. Bright mid-infrared photoluminescence from thin-film black phosphorus. *Nano letters* **19**, 1488-1493 (2019).
10. Huang, Shenyang, et al. From Anomalous to Normal: Temperature Dependence of the Band Gap in Two-Dimensional Black Phosphorus. *Physical Review Letters* **125**, 156802 (2020).
11. Morita, A. Semiconducting black phosphorus. *Applied Physics A* **39,** 227-242 (1986).
12. Li, Likai, et al. Direct observation of the layer-dependent electronic structure in phosphorene. *Nature nanotechnology* **12**, 21-25 (2017).
13. Thomsen, C., et al. Coherent phonon generation and detection by picosecond light pulses. *Physical review letters* **53**, 989 (1984).
14. Thomsen, C., et al. Surface generation and detection of phonons by picosecond light pulses. *Physical Review B* **34**, 4129 (1986).
15. Ge, Shaofeng, et al. Coherent longitudinal acoustic phonon approaching THz frequency in multilayer molybdenum disulphide. *Scientific reports* **4**, 1-7 (2014).
16. Vinod, M., G. Raghavan, and V. Sivasubramanian. Fano resonance between coherent acoustic phonon oscillations and electronic states near the bandgap of photoexcited GaAs. *Scientific reports* **8**, 1-9 (2018).
17. Meng, Shengjie, et al. Anisotropic charge carrier and coherent acoustic phonon dynamics of black phosphorus studied by transient absorption microscopy. *The Journal of Physical Chemistry* C **123**, 20051-20058 (2019).
18. Kôzuki, Yasushi, et al. Measurement of ultrasound velocity in the single crystal of black phosphorus up to 3.3 GPa gas pressure. *Journal of the Physical Society of Japan* **60**, 1612-1618 (1991).
19. Wang, Xiaomu, and Shoufeng Lan. Optical properties of black phosphorus. *Advances in Optics and photonics* **8**, 618-655 (2016).
20. Aivazian, Grant, et al. Many-body effects in nonlinear optical responses of 2D layered semiconductors. *2D Materials* **4**, 025024 (2017).
21. Ruppert, Claudia, et al. The role of electronic and phononic excitation in the optical response of monolayer WS2 after ultrafast excitation. *Nano letters* **17**, 644-651 (2017).
22. Trovatello, Chiara, et al. The ultrafast onset of exciton formation in 2D semiconductors. *Nature communications* **11**, 1-8 (2020).
23. Li, Diao, et al. Polarization and thickness dependent absorption properties of black phosphorus: new saturable absorber for ultrafast pulse generation. *Scientific reports* **5**, 1-9 (2015).
24. Xia, Fengnian, Han Wang, and Yichen Jia. Rediscovering black phosphorus as an anisotropic layered material for optoelectronics and electronics. *Nature communications* **5**, 1-6 (2014).
25. Lan, Shoufeng, et al. Visualizing optical phase anisotropy in black phosphorus. *ACS Photonics* **3**, 1176-1181 (2016).

26. Cartz, L., et al. Effect of pressure on bonding in black phosphorus. *The Journal of Chemical Physics* **71**, 1718-1721 (1979).
27. Henry, Laura, et al. Anisotropic thermal expansion of black phosphorus from nanoscale dynamics of Phosphorene layers. *Nanoscale* **12**, 4491-4497 (2020).
28. Yuan, Hongtao, et al. Polarization-sensitive broadband photodetector using a black phosphorus vertical p–n junction. *Nature nanotechnology* **10**, 707-713 (2015).
29. Stephenson, C. C., et al. The thermodynamic properties of elementary phosphorus The heat capacities of two crystalline modifications of red phosphorus, of α and β white phosphorus, and of black phosphorus from 15 to 300 K. *The Journal of Chemical Thermodynamics* **1**, 59-76 (1969).
30. Montanaro, Angela, et al. Visible pump–mid infrared pump–broadband probe: Development and characterization of a three-pulse setup for single-shot ultrafast spectroscopy at 50 kHz. *Review of Scientific Instruments* **91**, 073106 (2020).
